# *IRS1‐* rs10498210 G/A and *CCR5*‐59029 A/G polymorphisms in patients with type 2 diabetes in Kurdistan

**DOI:** 10.1002/mgg3.631

**Published:** 2019-03-18

**Authors:** Fatemeh Keshavarzi, Shadi Golsheh

**Affiliations:** ^1^ Department of Biology Sanandaj Branch Islamic Azad University Sanandaj Iran; ^2^ Department of Biology Kurdistan Science and Research Branch Islamic Azad University Sanandaj Iran

**Keywords:** *CCR5*, *IRS1*, PCR‐RFLP, polymorphism, type 2 diabetes

## Abstract

**Background:**

The insulin receptor substrate 1 (*IRS1*) is a critical factor in the signaling pathway for insulin, and mutations in this gene have been reported, which contribute to the ability to develop type 2 diabetes. The polymorphisms in the promoter region of C‐C motif chemokine receptor5 (*CCR5*) are also being studied as candidates for susceptibility to develop type 2 diabetes. The aim of the current study was to determine the relationship between *IRS1* and *CCR5* polymorphisms with type 2 diabetes in the Kurdistan population.

**Methods:**

Genomic DNA was isolated from the blood by salt extraction method and the polymorphisms were examined using Restriction Fragment Length Polymorphism (RFLP) method.

**Results:**

The results of current study indicated that the frequency of AA genotype in type 2 diabetic patients in both *CCR5* (OR = 2.9, *p* = 0.04) and *IRS1* (OR = 3.3, *p* = 0.036) were significantly more than controls.

**Conclusion:**

According to the results of this study, the presence of AA genotype in both *CCR5* and *IRS1* is associated with type 2 diabetes. There was no significant association between AG or GG genotypes with type 2 diabetes.

## 1 INTRODUCTION

Today, more than 265 million people are affected across the world. It is estimated that by the year 2030 this number will reach 366 million people (about 4/4 percent of the world's population), and now the cause of death is more than 1.1 million per year (including 50% of the population under‐70 years of age and 55% of women). On the other hand, given its negative effect on the economic growth of developing countries, it calls for universal mobilization to combat this disease (Bhattacharya, Dey, & Roy, 2007). Diabetes or diabetes mellitus is referred to as a heterogeneous group of metabolic disorders characterized by chronic hyperglycemia and carbohydrate, fat and protein metabolism disorders that result from a defect in the secretion of insulin, or impairment in its function, or both. Types of diabetes mellitus include type 1, type 2 diabetes and other kind of diabetes, but the two most common types of diabetes mellitus are type 1 and type 2, which are different in several aspects (Meshkani, Taghikhani, Mosapour et al., 2007). Type 1 diabetes has been identified with autoimmune destruction of pancreatic beta cells (insulin secreting cells) and accounts for about 5% of all diabetic people, while type 2 diabetes is a predominant disorder characterized by insulin resistance or a relative decline in insulin production, and accounts for about 90% of all types of diabetes mellitus (Meshkani, Taghikhani, Al‐Kateb et al., 2007). Important factors that predispose a person to type 2 diabetes are multifactorial, including genetic factors and environments. However, its inheritance has certainly not been proven, but it is believed that first–degree relatives of diabetic patients have a higher chance to develop the disease. In this regard, recognizing gene polymorphisms of this disease seems to be necessary (Häring et al., 2014). Multiple genes have been studied in the pathogenesis of type 2 diabetes.

One of these genes associated with type 2 diabetes is the insulin receptor substrate 1 (*IRS1,* OMIM association number, 147545) (Alharbi, Khan, Abotalib, & Al‐Hakeem, 2014; Alharbi, Khan, Munshi et al., 2014; Brender et al., 2013; Brunetti, Chiefari, & Foti, 2014) and another is the C‐C motif chemokine receptor5(*CCR5,* OMIM association number, 601373) (Balistreri et al., 2007; Mokubo et al., 2006; Muntinghe et al., 2009).

Insulin initiates a wide range of growth and metabolic effects by binding to its receptor and activating the property of tyrosine kinase. These events cause phosphorylation of tyrosine kinase residues at the level of anchored proteins, which include insulin receptor substrate proteins (*IRS1*) (White, 1997). The phosphorylated IRS proteins are used as multi–position anchored proteins for different molecules that have homologous domains (SH2) or Src. The activity of these second SH proteins triggers the signaling cascade and results in the activity of several downstream filters that ultimately transmit the insulin message to the cellular vector pathways, thereby regulating cell differentiation, growth, survival and metabolism. In different studies, the frequency of *IRS1* polymorphisms in type 2 diabetic patients was more than control group (Chang et al., 2015; Huri, Makmor‐Bakry, Hashim, Mustafa, & Ngah, 2012; Richter & Hargreaves, 2013). The *IRS1* is a cytoplasmic substrate for insulin and also is a receptor for *IGF‐1* (OMIM association number, 147440), which plays a vital role in signaling. In recent studies, various roles of *IRS1* have been discovered, especially in patients with noninsulin diabetes mellitus. The *IRS1* gene polymorphisms were identified in 1993 (Audouze, Brunak, & Grandjean, 2013; Audouze et al., 2013).

Chemokines are a large family of low molecular weight secretion proteins that play fundamental roles in physiological and pathophysiological processes such as angiogenesis, inflammation, atherosclerosis, and autoimmune or allergic or infectious diseases (Herder et al., 2005; Ullrich, 2015). Their initial function is to regulate the migration of leukocytes at the concentration gradient, but they also play a role in the activation of the cells producing and secreting inflammatory mediators. These chemokines do their function by connecting to their G‐protein receptors (Herder et al., 2005). Excessive nutrition that has high levels of glucose and fatty acids can put a stress on the pancreatic islets and insulin–sensitive tissues such as fat and liver and muscle, leading to the production and release of topical cytokines and inflammatory chemokines (Baggiolini, 2001). Among these inflammatory chemokines *CCL2*(OMIM association number, 158105)*, MIP* (OMIM association number, 154050), *CCL5* (OMIM association number, 187011), and *CCL8*(OMIM association number, 602283) are mentioned. These chemokines interact with their receptors, triggering monocytes, as well as increasing the number of macrophages in the inflammation position. Chemokine receptors that can be mentioned include CC chemokine receptor type 2 (*CCR2,* OMIM association number, 601267), *CCL2* and also chemokine receptors *CCR5, MIP, CCL5* and *CCL8* (Ahluwalia et al., 2009).

*CCR5* is located at 3q21.3 position on the chromosome. The *CCR5*‐59029A/G polymorphism has been reported in the promoter region of the *CCR5* receptor gene (Abbas, Lichtman, & Pillai, 2014). Studies indicated that the *CCR5*‐59029 A/G genotype results in increased expression of this receptor by peripheral blood mononuclear cells of individuals with this genotype, and therefore it is probably the genotype regulating the expression of *CCR5* (Baggiolini, 2001; Mokubo et al., 2006).

In this regard, the relationship between the *IRS1*‐rs10498210 G/A polymorphisms and *CCR5‐*59029 A/G and the risk of type 2 diabetes have not been clearly and precisely indicated. Therefore, this study was conducted with the aim of investigating this relationship.

## 2 METHODS

### 2.1 Sampling method

This research is a case–control study. The study was ethically approved by the regional Ethics Committee of Sanandaj Branch, Islamic Azad University. During this study, the peripheral blood samples of type 2 diabetic patients (fasting blood glucose higher than 150 mg/dl in two times) and nondiabetic subjects as control (fasting blood glucose less than 100 mg/dl in two times) were collected in tubes containing anticoagulant EDTA (K2). Patients were selected randomly among individuals who were referred to the Kurdistan Diabetes Center in Sanandaj. Patients were selected in such a way that their diabetes was controlled (measured by HbAlc by the diabetes center). Inclusion criteria were diagnosed according to the American Diabetes Association diagnostic criteria (the blood glucose level of >250 mg/dl or severe hyperglycemia). Written consent was received from the individuals and they were informed that sampling was for research purposes only.

### 2.2 DNA extraction

Extraction of DNA from the blood samples was performed using salt extraction method and DNA extraction was performed using 1% agarose gel. The isolated DNA was placed in separate microtubes and stored at −24°C until PCR was performed.

### 2.3 Genotypes determination

Determination of genotype was carried out using PCR‐RFLP method and the primers presented in Table 1 were used for replication of fragment. For the *CCR5‐59029 A/G* (its GenBank reference is NC_000003.12) polymorphism, the primers were taken from other articles but for *IRS1‐rs10498210 G/A* (its GenBank reference is NC_000002.12) polymorphism were designed.

**Table 1:** Primers

*IRS1*‐rs10498210G/A	*CCR5*‐59029A/G	
5′‐ ACAGCCAAAAGGTAAAGCGT ‐3′	5′‐CCCGTGAGCCCATAGTTAAAACTC‐3′	Forward
5′‐ CCCTTCTCAAAGTACAGCATGT ‐3′	5′‐TCACAGGGCTTTTCAACAGTAAGG‐3′	Reverse
bp371	bp258	Product size

*Note*. The GenBank reference of IRS1 NC_000002.12.

The GenBank reference of CCR5 NC_000003.12.

PCR was performed with a final volume of 20 μl using Sinagene PCR kit. The PCR cycles of the desired gene are presented in Table 2 separately. To ensure correct replication of the desired piece, the PCR products were loaded onto 1.8% agarose gel and its quality was determined.

**Table 2:** PCR proliferation conditions

Genes	Cycling condition				
	Initial denaturation	Denaturation	Annealing	Extension	Final extension
*CCR5*	94°C	94°C	60°C	72°C	72°C
	4 min	30 s	60 s	60 s	5 min
Repeated for 34 cycles					
*IRS1*	94°C	94°C	60°C	72°C	72°C
	4 min	30 s	60 s	60 s	5 min
Repeated for 34 cycles					

*Note*. The GenBank reference of *IRS1* NC_000002.12.

The GenBank reference of CCR5 NC_000003.12.

In order to cut the desired region in the *CCR5* gene, the SduI enzyme was selected, which is detected as GGGCAC, and consequently, in the presence of the allele G in polymorphic position, enzyme cut the piece and, in the presence of the allele A. The piece produced by PCR for *CCR5* is a 258 base pair piece, and if the piece is cut, two fragment of 131 and 127 bases are created. To cut the desired region in *IRS1*, the MaeII enzyme was selected, which was detected as ACGT. The PCR–proliferated piece is 371 bp. In the presence of the allele G in the polymorphic position, the enzyme had cut position, which resulted in two fragment of 229 and 142 bp size, and in the presence of the allele A at the polymorphic position, the 371 bp piece was not broken. Then the digested products were loaded on a 3% agarose gel and their genotypes were determined.

### 2.4 Statistical analysis

Data analysis was performed using software popgene1.32 and SPSS v20 and at significant level (*p* < 0.05).

## 3 RESULTS

**Figure 1:**
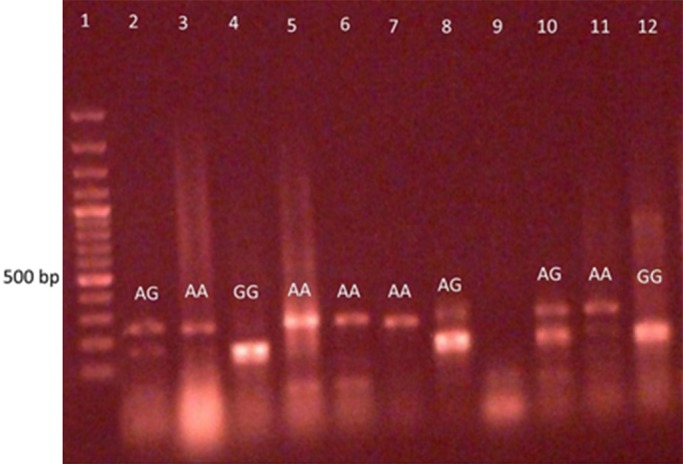
Genotype Detection *CCR5*‐59029A/G (NG_012637.1) The piece produced by PCR for *CCR5* is a 258 base pair piece, and if the piece is cut, two fragment of 131 and 127 bp size are created. In the presence of the allele G in polymorphic position, the enzyme cut the piece and, in the presence of the allele A, the piece does not cut

This case–control study was performed on 190 unrelated individuals, including 120 patients with type 2 diabetes and 70 healthy controls. Figure 1 shows the image of the 3% agarose gel for *CCR5‐*59029 A/G polymorphism and also shows how to determine its genotype in comparison to the ladder and fragment size as described above.

In the population studied, the frequency of genotypic polymorphism in *CCR5* was as follows: among the 120 patients, the frequency of GG, AG and AA genotypes was 0.375, 0.442 and 0.183, respectively, and among the 70 control subjects, the frequency of GG, AG, and AA genotypes was 0.428, 0.5 and 0.071, respectively.

**Figure 2:**
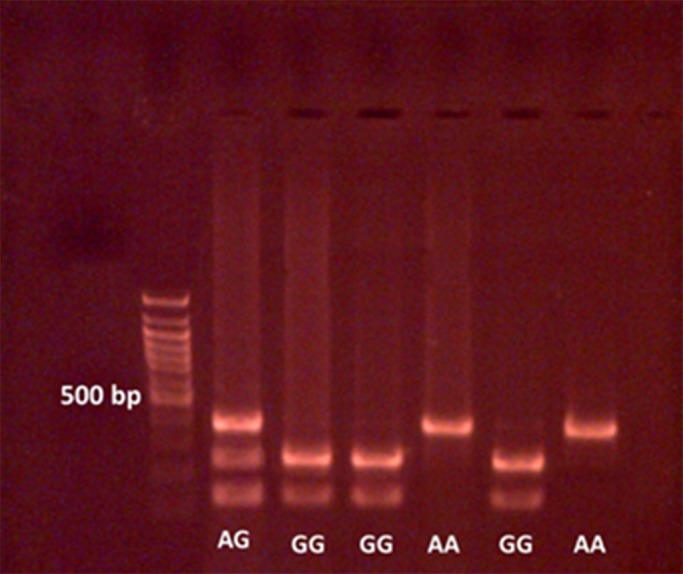
Genotype Detection *IRS1*‐ rs10498210 G/A (NG_015830.1) The PCR–proliferated piece is 371 bp. In the presence of the allele G in the polymorphic position, the enzyme has cut position, resulting in two fragment of 229 and 142 bp, and in the presence of the allele A at the polymorphic position, the 371 bp piece is not broken, and totally one piece will remain

Figure 2 shows the image of the 3% agarose gel for rs10498210 and also shows how to determine its genotype in comparison to the ladder and fragment size as descriptive above.

In the studied population, the frequency of genomic polymorphism in *IRS1* was as follows: among the 120 patients, the frequency of GG, AG, and AA genotypes was 0.533, 0.35 and 0.116, respectively, and also, among the 70 control subjects, the frequency of GG, AG and AA genotypes was 0.714, 0.271 and 0.014, respectively.

In this study, Hardy–Weinberg equilibrium and heterozygosity were also studied for populations. The Hardy–Weinberg equilibrium points to the fact that the genetic and genotype frequency is constant from generation to generation. The probability level in both, type 2 diabetic and control subjects was greater than 0.05 for *IRS1* and *CCR5*, which indicates a Hardy–Weinberg equilibrium in these populations.

Heterozygosity for a gene position is defined as a frequency of heterozygote people for that position relative to the total population. For a gene position, if the heterozygosity is greater than 0.1, it is polymorphic and if it is more than 0.7, it is extremely Polymorphic. Based on the results of this study, it was found that the difference between the observed and expected heterozygosity for both studied polymorphisms was less than 0.1, so the gene positions in this study are not polymorphic. In the next step, the mean of patient's clinical data which were collected from their profiles from the Diabetes Center of Kurdistan was analyzed. The results of clinical data analysis are presented in Tables 3 and 4.

**Table 3:** Comparison of type 2 diabetic patient's clinical data among different genotypes of polymorphism IRS1‐ rs10498210 G/A

AG‐GG	AA‐GG	AA‐AG	Clinical data
0.196	0.107	0.536	Weight
0.995	0.633	0.700	Systolic blood pressure
0.154	0.027	0.203	Diastolic blood pressure
0.322	0.057	0.447	Total cholesterol
0.532	0.443	0.958	Triglyceride
0.428	0.000	0.000	Cholesterol HDL
0.277	0.098	0.288	Cholesterol LDL
0.924	0.029	0.016	Fasting blood glucose
0.428	0.453	0.768	HBA1C

*Note*. The GenBank reference of IRS1 NC_000002.12.

**Table 4:** Comparison of type 2 diabetic patient's clinical data among different genotypes of polymorphism CCR5‐59029A/G

AG‐GG	AA‐GG	AA‐AG	Clinical data
0.664	0.075	0.156	Weight
0.000	0.211	0.086	Systolic blood pressure
0.540	0.867	0.079	Diastolic blood pressure
0.263	0.000	0.000	Total cholesterol
0.061	0.771	0.219	Triglyceride
0.102	0.077	0.536	Cholesterol HDL
0.430	0.000	0.000	Cholesterol LDL
0.000	0.000	0.220	Fasting blood glucose
0.798	0.456	0.201	HBA1C

*Note*. The GenBank reference of CCR5 NC_000003.12.

Also weight, Systolic blood pressure, Diastolic blood pressure, Total Cholesterol, Triglyceride, cholesterol HDL, cholesterol LDL, Fasting blood Glucose and HBA1C were significantly higher in the patients group when compared to the control group (Tables 3 and 4).

## 4 DISCUSSION

Achieved frequencies significance between type 2 and control subjects were statistically analyzed using SPSS v20 software. Concerning the *CCR5* gene polymorphism, there was a significant difference between the presence of AA genotype in the control and diabetic groups (OR = 2.9, *p* = 0.04). Also, for *IRS1* gene polymorphism, there was a significant difference between the presence of AA genotype in these two groups (OR = 3.3, *p* = 0.036).

McDermott et al., who found for the first time the A/G polymorphism in the 59029 base pair in the promoter region of this gene, reported that both alleles of this polymorphism are common in societies and the allelic frequency of 59029A depending on the ethnic population varies between 43% and 68%. Differences in the frequency of allelic A in different communities can be due to genetic differences between populations (Abbas et al., 2014; McDermott et al., 1998). According to the results, it is possible that the *CCR5* genotype (AA 59029) plays an important role in the pathogenesis of type 2 diabetes. Studies indicated that the *CCR5*‐59029A/‐ genotype results in increased expression of *CCR5* by peripheral blood mononuclear cells of individuals with this genotype. In a study by Dytfeld et al. (2006), the expression of *CCR5* receptor expression was measured on the peripheral blood mononuclear cells of type 2 diabetics, and it was determined that the expression of *CCR5* receptor on the cell surface in Type 2 diabetic patients was also increasing, and high expression of this receptor can be considered as an indicator of atherosclerosis in diabetic people (Passam et al., 2005). Given the evidence of Type 2 diabetes which was recently provided and Type 2 diabetes introduced as an inflammatory disease, it can be expected that high expression of this receptor (*CCR5*) on the level of single–cellular cells of the blood increases inflammatory responses and increases the risk of type 2 diabetes. However, in order to confirm with certainty, the existence of such a connection, further studies in a wider population are needed. Regarding the role of *IRS1* in the pathway of insulin signaling and the negative effect of rs10498210 polymorphism on the performance of this protein, it can be expected that this polymorphism is present in the etiology of type 2 diabetes. Recent studies have indicated that *IRS1* plays an important role in regulating insulin secretion in beta cells of the pancreas. It has been shown that glucose–stimulated insulin secretion may be triggered by the autocrine activation of the insulin signaling pathway, including insulin receptor phosphorylation, tyrosine phosphorylation in *IRS1*, and the activation of *PI3‐Kinase*. Putting together these data leads to the hypothesis that a single molecular impairment in the pathway of insulin signaling, including an incomplete interaction between *PIK3CA*(OMIM association number, 171834) and *IRS1*, may lead to insulin resistance, as well as insulin secretion defect. So far, there has been a weak link between this polymorphism and type 2 diabetes, especially in obese people, but few studies have reported the association between this polymorphism and diabetes. In general, a variety of allele A in *IRS1* frequencies have been reported in many studies, and controversial reports have revealed the association of this polymorphism with type 2 diabetes (Yousef et al., 2018). Finally, according to the results of this study, it can be concluded that the probability of the positive effect of allele A on the studied polymorphisms *IRS1‐rs10498210 G/A* and *CCR5*‐59029 A/G increases the risk of type 2 diabetes. Also, clinical data from diabetic patients suggest that the allele A form both the studied polymorphisms play a positive role in increasing the risk of cardiovascular disease in type 2 diabetic patients. However, to be sure about the impact of these polymorphisms on type 2 diabetes, it is necessary to conduct a study on a larger population. It is also possible to compare the clinical data of patients with healthy subjects and examine the effect of these two polymorphisms on the clinical data of these two groups and more effectively to study the role of these polymorphisms in increasing the risk of disease cardiovascular disease. The two studied genes in current study are associated with insulin resistance based on two different mechanisms. *IRS1* plays a role in the insulin signaling pathway in its target tissues and *CCR5* plays a role in the inflammation pathway in fatty tissues and beta cells in the pancreas. By simultaneous examination of these two genes and the effect of their different variants together, in type 2 diabetic patients, greater recognition of the importance of each of these pathways in the pathogenesis of type 2 diabetes can be obtained.

## 5 CONCLUSION

According to the results of this study, the presence of AA genotype in both *CCR5* and *IRS1* is associated with type 2 diabetes. There was no significant association between AG or GG genotypes with type 2 diabetes. Due to the nature of the type 2 diabetes mellitus and the role of the environmental factors involved in it, the genetic screening of individuals in a population can be performed by identifying the genes and varieties involved in the disease, and as a result, individuals and populations with high risk for infection can be Identified and controlled more and more risky environmental factors, such as obesity in these populations.

## CONFLICT OF INTEREST

No.

## AUTHOR CONTRIBUTION

Fatemeh keshavarzi designed the study and wrote the paper; Shadi Golsheh, conceived the experiments, prepared the figures; collected the samples. All authors gave final approval for the manuscript to be submitted for publication.

## ETHICS STATEMENT

The study was ethically approved by the regional Ethics Committee of Sanandaj Branch, Islamic Azad University.
